# CircNFIX promotes progression of glioma through regulating miR-378e/RPN2 axis

**DOI:** 10.1186/s13046-019-1483-6

**Published:** 2019-12-30

**Authors:** Chenyu Ding, Zanyi Wu, Honghai You, Hongliang Ge, Shufa Zheng, Yuanxiang Lin, Xiyue Wu, Zhangya Lin, Dezhi Kang

**Affiliations:** Department of Neurosurgery, The First Affiliated Hospital of Fujian Medical University, 20 Chazhong Road, Taijiang District, Fuzhou, 350001 Fujian China

**Keywords:** Glioma, circNFIX, miR-378e, RPN2

## Abstract

**Background:**

Circular RNA nuclear factor I X (circNFIX) has been reported to play an important role in glioma progression. However, the mechanism by which circNFIX participates in glioma progression remains poorly understood.

**Methods:**

GERIA online were used to analyze the abnormally expressed genes in glioma tissues. The expression levels of circNFIX, microRNA (miR)-378e and Ribophorin-II (RPN2) were measured by quantitative real-time polymerase chain reaction or western blot. Cell cycle distribution, apoptosis, glycolysis, migration and invasion were determined by flow cytometry, special kit and trans-well assays, respectively. The target association between miR-378e and circNFIX or RPN2 was confirmed by luciferase reporter assay, RNA immunoprecipitation and pull-down. Xenograft model was established to investigate the role of circNFIX in vivo.

**Results:**

The expression of circNFIX was enhanced in glioma tissues and cells compared with matched controls and high expression of circNFIX indicated poor outcomes of patients. Knockdown of circNFIX led to arrest of cell cycle, inhibition of glycolysis, migration and invasion and promotion of apoptosis in glioma cells. circNFIX was a sponge of miR-378e. miR-378e overexpression suppressed cell cycle process, glycolysis, migration and invasion but promoted apoptosis. miR-378e silence abated the suppressive role of circNFIX knockdown in glioma progression. RPN2 as a target of miR-378e was positively regulated via circNFIX by competitively sponging miR-378e. Silencing circNFIX decreased glioma xenograft tumor growth by regulating miR-378e/RPN2 axis.

**Conclusion:**

Knockdown of circNFIX inhibits progression of glioma in vitro and in vivo by increasing miR-378e and decreasing RPN2, providing a novel mechanism for understanding the pathogenesis of glioma.

## Background

Glioma is a nervous system tumor with high mortality and the current treatment is surgery combined with radiotherapy or chemotherapy [1]. Despite many advances in the treatment of glioma, the effective strategies remain limited. Hence, it is imperative to develop new targets for improving cancer treatment. One promising therapy is based on circular RNAs (circRNAs).

circRNAs are a member of noncoding RNAs playing essential roles in various cancers through regulating multiple biological processes, including proliferation, apoptosis, cell cycle process, migration and invasion [2], which are circularized by joining the 3′ end of the RNA to the 5′ end [3]. They are distributed in neuronal tissues and exhibit key functions in brain diseases, including glioma [4, 5]. For instance, Lei et al. reported that circRNA hsa_circ_0076248 promotes proliferation and invasion of glioma by regulating miR-181 and silent information regulator 1 (SIRT1) [6]. Shi et al. showed that hsa_circ_0014359 contributes to glioma progression by targeting miR-153/phosphatidylinositol 3 kinase (PI3K) signaling [7]. Furthermore, Wang et al. suggested that hsa_circ_0005198 promotes proliferation, migration and invasion of glioma cells by sponging miR-1294 [8]. Apart from these, hsa_circ_0079593 has also been reported to facilitate cancer development and indicate poor prognosis in glioma [9]. As for nuclear factor I X (NFIX), it has been demonstrated to be implicated in the development of multiple organ systems, including brain [10, 11]. More importantly, the corresponding circRNA NFIX (circNFIX) could promote cell proliferation in glioma by regulating miR-34a-5p [12]. However, the influence and mechanism of circNFIX in glioma need more researches.

microRNAs (miRNAs) as a class of small noncoding RNAs have been regarded as important targets for the therapy of brain cancer [13]. Moreover, emerging evidence suggested that miRNAs play pivotal roles in tumorigenesis and progression of glioma [14]. Previous studies revealed that miR-378 is abnormally expressed and plays a tumor suppressive role in glioma [15, 16]. miR-378e is an important member of miR-378 family and this study wants to investigate the greater depth role of this miRNA in glioma. Previous study suggested that circRNAs exhibit major biological roles through the networks of competing endogenous RNAs (ceRNAs) in cancers [17]. The database of starBase online predicted the complementary sequences between miR-378e and circNFIX/Ribophorin-II (RPN2), suggesting the potential ceRNA network of circNFIX/miR-378e/RPN2. In this study, we focused on the function and mechanism of circNFIX in glioma cells. By combining in vitro and in vivo experiments, we confirmed that the regulatory mechanism of circNFIX was associated with miR-378e/RPN2 axis in glioma.

## Materials and methods

### Gene expression analysis

GERIA online (http://gepia.cancer-pku.cn/) was used to analyze the abnormally expressed mRNAs in glioma tissues. The expression of RPN2 in glioma tissues was expressed as a boxplot (Fig. 7b), which displays the information about the central tendency, symmetry, skew and outliers and is plotted following the instruction [18].

### Patient samples and cell culture

Sixty-four patients with glioma and 15 patients with cerebral trauma as controls were recruited from Department of Neurosurgery, the First Affiliated Hospital of Fujian Medical University. The corresponding brain tissues were collected by surgery and stored at -80 °C.The informed consents of all participants were obtained before this study and this study was approved by the Ethics Committee of Department of Neurosurgery, the First Affiliated Hospital of Fujian Medical University.

The human astrocyte cell line (HA) and glioma cell lines (T98, U251, SW1783 and A172) were obtained from BeNa Culture Collection (Beijing, China). All cells were cultured in DMEM (Sigma, St. Louis, MO, USA) containing 10% fetal bovine serum at 37 °C with 5% CO_2_.

### Cell transfection

The overexpression vector of circNFIX (hsa_circ_0049658) was generated on the basis of pcDNA3.1 vector (Thermo Fisher Scientific, Wilmington, DE, USA) and pcDNA3.1 empty vector (pcDNA) was used as a control. siRNA targeted circNFIX (si-circNFIX) (5′-CACCGGACAGAAUCCGGACAA-3′), siRNA negative control (si-NC) (5′-UUCUCCGAACGUGUCACGUTT-3′); miR-378e mimic (miR-378e) (5′-ACUGGACUUGGAGUCAGGA-3′), miRNA negative control (miR-NC) (5′-UUCUCCGAACGUGUCACGUTT-3′), miR-378e inhibitor (anti-miR-378e) (5′-UCCUGACUCCAAGUCCAGU-3′) and inhibitor negative control (anti-miR-NC) (5′-CAGUACUUUUGUGUAGUACAA-3′) were generated by GenePharm (Shanghai, China). These oligonucleotides with a final concentration of 40 nM or vectors were transfected into T98 and U251 cells by using Lipofectamine 2000 (Thermo Fisher Scientific) for 24 h.

### Trans-well assay

Cell migration was performed using Trans-well chamber and cell invasion was detected using chamber with Matrigel. Transfected T98 and U251 cells (1 × 10^5^/well) were suspended in DMEM without serum in the upper chambers and 500 μl DMEM medium with 10% FBS was added into the lower chambers. After the incubation for 24 h, the cells adhering to the under layer of the membranes were stained with 0.1% crystal violet. The number of migrated or invasive cells was counted with three random fields under a 200× magnification microscope.

### Flow cytometry

Transfected T98 and U251cells were cultured for 48 h. For analysis of cell cycle distribution, cells were washed and fixed with 75% ethanol (Sigma). Then cells were incubated with RNase A and PI solution for 20 min at 37 °C. For apoptosis analysis, cells were lysed in binding buffer and then stained with Annexin V-FITC and PI in Annexin V-FITC Apoptosis Detection Kit (Beyotime, Shanghai, China) for 15 min in the dark. The distribution of cell cycle and apoptosis were analyzed with a flow cytometer.

### Glucose consumption and lactate production

After the indicated transfection, T98 and U251 cells were cultured in 96-well plates for 48 h. Then cells were washed and collected for analyses of glucose consumption using Glucose Uptake Colorimetric Assay Kit (Sigma) and lactate production using Lactate Assay Kit (Sigma) according to the manufacturer’s instructions. The concentrations of glucose and lactate were analyzed according to the absorbance with a microplate reader (Bio-Rad, Hercules, CA, USA) and normalized to total protein detected by BCA Kit (Vazyme, Nanjing, China).

### Quantitative real-time polymerase chain reaction(qRT-PCR)

The RNA was extracted from tissues or cells using Trizol reagent (Thermo Fisher Scientific) according to the manufacturer’s instructions and quantified by NanoDrop 2000 spectrophotometer (Thermo Fisher Scientific). To improve the purity of circRNAs, the RNA was treated by RNase R (Geneseed, Guangzhou, China). The cDNA was generated with 500 ng RNA using TaqMan miRNA Reverse Transcription Kit (Applied Biosystems, FosterCity, CA, USA) or Prime-Script RT reagent kit (TaKaRa, Dalian, China) following the manufacturer’s instructions. The qRT-PCR was performed using SYBR mix (TaKaRa) on CFX96 Real-time PCR Systems (Bio-Rad). The primer sequences used in this research were listed as: circNFIX (Forward, 5′-AGGAGATGCGGACATCAAAC-3′; Reverse, 5′-GTGAAATACGGGCTCGACTG-3′); RPN2 (Forward, 5′- AGGAAGTGGTGTTTGTTGCC-3′; Reverse, 5′-ACAGTCGAGGGAGCTTCTTC-3′); miR-378e (Forward, 5′- GGGACTGGACTTGGAGTCA-3′; Reverse, 5′-GTGCGTGTCGTGGAGTCG-3′); GAPDH (Forward, 5′-GAATGGGCAGCCGTTAGGAA-3′; Reverse, 5′-AAAAGCATCACCCGGAGGAG-3′); U6 (Forward, 5′-CTCGCTTCGGCAGCACA-3′; Reverse, 5′-AACGCTTCACGAATTTGCGT-3′). GAPDH and U6 were regarded as internal control for circNFIX, RPN2 or miR-378e, respectively (Additional file 1: Supplementary materials and methods). The relative expression levels of circNFIX, miR-378e and RPN2 mRNA were analyzed by 2^-ΔΔCt^ method [19].

### Western blot

The proteins were extracted from cells or tissues using RIPA buffer (Beyotime) with protease inhibitors. The BCA Kit was applied to the detection of protein concentrations. Then equal amounts (20 μg) of proteins denatured by boiled water bath were separated by SDS-PAGE and then transferred onto 0.45 μm PVDF membranes (Millipore, Billerica, MA, USA). The membranes were blocked in TBST with 5% non-fat milk for 1 h and then incubated with rabbit anti-human primary antibodies against HK2 (ab227198, 1:5000 dilution, Abcam, Cambridge, MA, USA), RPN2(ab244399, 1:2000 dilution, Abcam) or β-actin(ab227387, 1:10000 dilution, Abcam) as a loading control at 4 °C overnight and goat anti-rabbit secondary antibody (ab97051, 1:10000 dilution, Abcam) at room temperature for 2 h. The signals were developed using ECL Kit (Beyotime) and the relative protein levels of HK2 and RPN2 were normalized to the control group.

### Luciferase reporter assay, RNA immunoprecipitation (RIP) and RNA pull-down

StarBase online predicted the potential targets of circNFIX or miR-378e. The sequences of circNFIX and 3’UTR sequences of RPN2 containing miR-378e complementary sites were cloned into pGL3-control luciferase reporter vectors (Promega, Madison, WI, USA) and named circNFIX-WT or RPN2-WT respectively. To mutate the putative seed sites, Q5 Site Directed Mutagenesis Kit (New England Biolabs, Ipswich, MA, USA) was used and the mutants were named as circNFIX-MUT or RPN2-MUT respectively. T98 and U251 cells were co-transfected with circNFIX-WT, circNFIX-MUT, RPN2-WT or RPN2-MUT, together with miR-378e or miR-NC. After 24 h post-transfection, luciferase reporter system (Promega) was used to assess the luciferase activity in T98 and U251 cells.

Magna RNA immunoprecipitation kit (Millipore) was used for RIP assay. T98 and U251 cells transfected with miR-378e or miR-NC were lysed in lysis buffer and then incubated with immunoprecipitation buffer containing magnetic beads coated with Ago2 antibody. Input and IgG were used as controls. The RNA in complex was extracted and the levels of circNFIX and RPN2 were detected by qRT-PCR.

For RNA pull-down assay, wild-type miR-378e, mutant miR-378e and negative control (NC) were labeled with biotin and incubated with streptavidin beads (Thermo Fisher Scientific) overnight at 4 °C. T98 and U251 cells were lysed and then cell lysates were incubated with bead-biotin complex for 2 h. Next, the RNA bound to the bead was extracted and used for qRT-PCR. The level of circNFIX was measured.

### Xenograft model

The animal experiments were performed in accordance with the guide for care and use of laboratory animals and this study was approved by the Ethics Committee of Department of Neurosurgery, the First Affiliated Hospital of Fujian Medical University. Thirty BALB/c nude mice (4-week-old, male) were purchased from Shanghai Animal Laboratory Center (Shanghai, China) and then randomly divided into six groups (*n* = 5) for xenograft model. T98 and U251 cells were infected with lentivirus expressing shRNA for circNFIX (sh-circNFIX, 5′-GCACUUAAGUUUCCAGGACUG-3′), negative control (sh-NC, 5′-GCUAGAACAGCAUGGUCCA-3′) or empty lentivirus for 24 h and the stably cells were selected by 1 μg/ml puromycin. 1 × 10^6^ stably transfected cells were injected subcutaneously into the right limbs of the mice. The tumor volume was measured every week and calculated as volume (mm^3^) = length × width^2^ × 0.5. After monitored for five weeks, mice were killed and tumor weight was detected, followed by further analyses of circNFIX, miR-378e and RPN2 protein levels in tumor tissues.

### Statistical analysis

The experiments were repeated at least three times. Data were expressed as mean ± standard deviation (S.D.) and difference of data in different groups was investigated by Student’s *t*-test or one-way ANOVA followed by Tukey’s test, processed by using GraphPad Prism 7 software (GraphPad Inc., La Jolla, CA, USA). Overall survival of patients was generated by Kaplan-Meier plot and assessed by log-rank test. The association between circNFIX level and clinicopathologic features of glioma patients was analyzed by χ2 test. *P* < 0.05 was considered significant.

## Results

### circNFIX expression is increased and indicates poor outcomes of patients in glioma

**Fig. 1:**
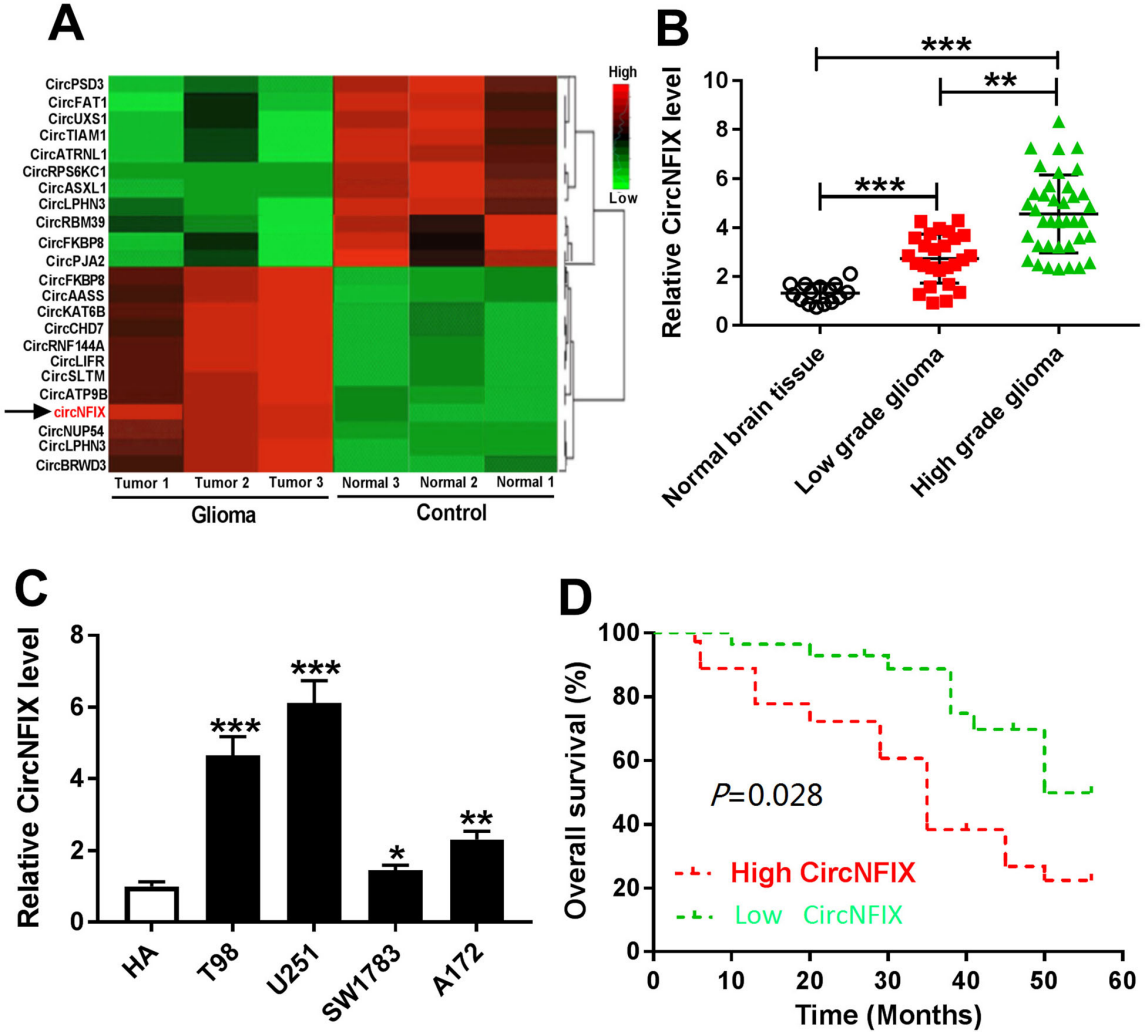
circNFIX expression is increased in glioma. (**a**) The heat map of expression levels of 23 circRNAs in glioma tissues. (**b**) qRT-PCR assay detected the level of circNFIX in low (*n* = 26) or high (*n* = 38) grade glioma group and normal tissues (*n* = 15). (**c**) qRT-PCR assay measured the abundance of circNFIX in glioma cells and normal astrocyte cell line. (**d**) Overall survival of glioma patients in high (*n* = 36) or low (*n* = 28) circNFIX group was analyzed. *P < 0.05, **P < 0.01, ***P < 0.001 compared with indicated control group

The data of heat map displayed 23 differentially expressed circRNAs including 11 down-regulated and 12 up-regulated circRNAs, of which circNFIX was significantly increased in glioma tissues (Fig. 1a). Moreover, we measured the level of circNFIX in 64 glioma tissues and 15 normal samples. As shown in Fig. 1b, the expression level of circNFIX was evidently enhanced in glioma tissues compared with that in normal control and there was a remarkable difference between low- and high-grade group. Furthermore, compared with that in HA cells, the abundance of circNFIX was notably up-regulated in glioma cells, especially in T98 and U251 cells (Fig. 1c). According to circBase, the spliced sequence length of circNFIX (hsa_circ_0049658) was 695 bp, which arose from NFIX gene, locates at chromosome 19 (13183860–13,192,669) and the back-spliced junction of hsa_circ_0049658 was validated by sanger sequencing (Additional file 2: Figure S1A). Unlike the linear RNA NFIX, circNFIX was more resistant to RNase R due to the looping characteristics (Additional file 2: Figure S1B and S1C). In addition, the 64 patients were divided into high or low circNFIX expression group and we found that high expression of circNFIX was associated with WHO stage, tumor size and poor survival of patients, but not with other clinical characteristics, including age, gender and tumor location (Table 1 and Fig. 1d).

**Table 1:** Correlations between circNFIX expression and clinical characteristics in glioma patients

Clinicopathologic features	N (%)	Relative CircNFIX level		*P* value
		High (%)	Low (%)	
Total cases	64 (100.0)	36 (56.3)	28 (43.7)	
Age (years)				0.7647
≥ 45	40 (62.5)	22 (55.0)	18 (45.0)	
< 45	24 (37.5)	14 (58.3)	10 (41.7)	
Gender				0.4363
Male	37 (57.8)	20 (54.1)	17 (45.9)	
Female	27 (42.2)	16 (59.3)	9 (40.7)	
WHO grade				0.0177
I-II	26 (40.6)	10 (38.5)	16 (61.5)	
III-IV	38 (59.4)	26 (68.4)	12 (31.6)	
Tumor size (cm)				0.0262
≥ 3	36 (56.3)	23 (63.9)	13 (36.1)	
< 3	28 (43.7)	13 (46.4)	15 (53.6)	
Tumor location				0.5239
Supratentorial	43 (67.2)	23 (53.5)	20 (46.5)	
Subtentorial	21 (32.8)	13 (61.9)	8 (38.1)	

### Knockdown of circNFIX inhibits progression of glioma

**Fig. 2:**
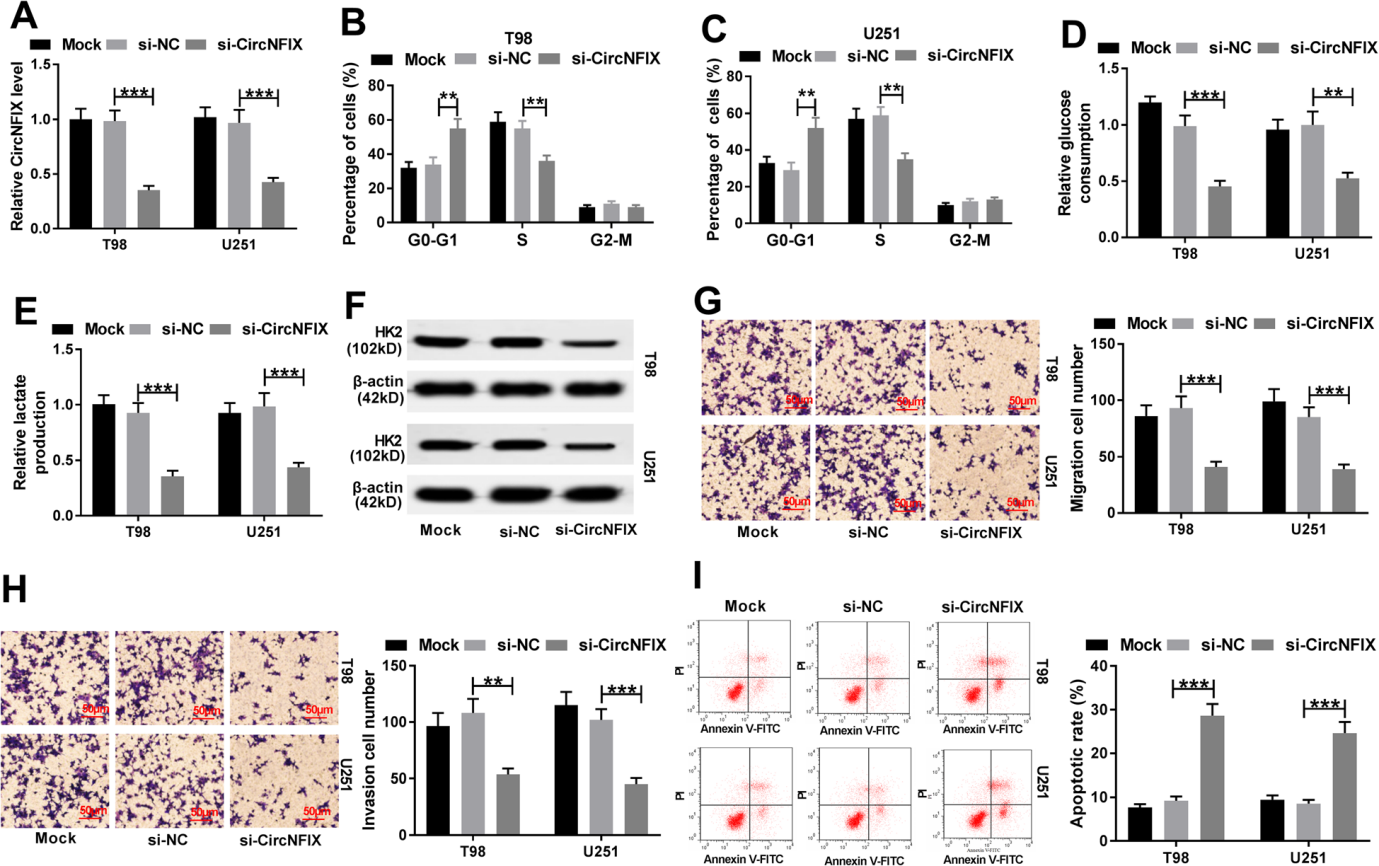
Knockdown of circNFIX induces cell cycle arrest and apoptosis and inhibits glycolysis, migration and invasion in glioma cells. (**a**) qRT-PCR assay determined the transfection efficacy in T98 and U251 cells after transfection of si-circNFIX or si-NC. Cell cycle distribution (**b** and **c**), glucose consumption (**d**), lactate production (**e**), HK2 protein level (**f**), migration (**g**), invasion (**h**) and apoptosis (**i**) were determined in T98 and U251 cells transfected with si-circNFIX or si-NC. Mock is non-transfected group. **P < 0.01, ***P < 0.001 compared with si-NC group

To investigate the role of circNFIX in glioma, the abundance of this circRNA in T98 and U251 cells was knocked down by using si-circNFIX (Fig. 2a). Moreover, data of flow cytometry displayed that knockdown of circNFIX led to cell cycle arrest at G0-G1 phase in T98 and U251 cells (Fig. 2b and c). In addition, silence of circNFIX significantly inhibited glycolysis in the two cell lines, revealed by reduction of glucose consumption, lactate production and HK2 protein level (Fig. 2d-f). Furthermore, analysis of trans-well described that the abilities of migration and invasion in T98 and U251 cells were markedly repressed by silencing circNFIX (Fig. 2g and h). Besides, results of flow cytometry also exhibited that circNFIX knockdown resulted in great apoptosis production in T98 and U251 cells (Fig. 2i).

### circNFIX is a sponge of miR-378e

**Fig. 3:**
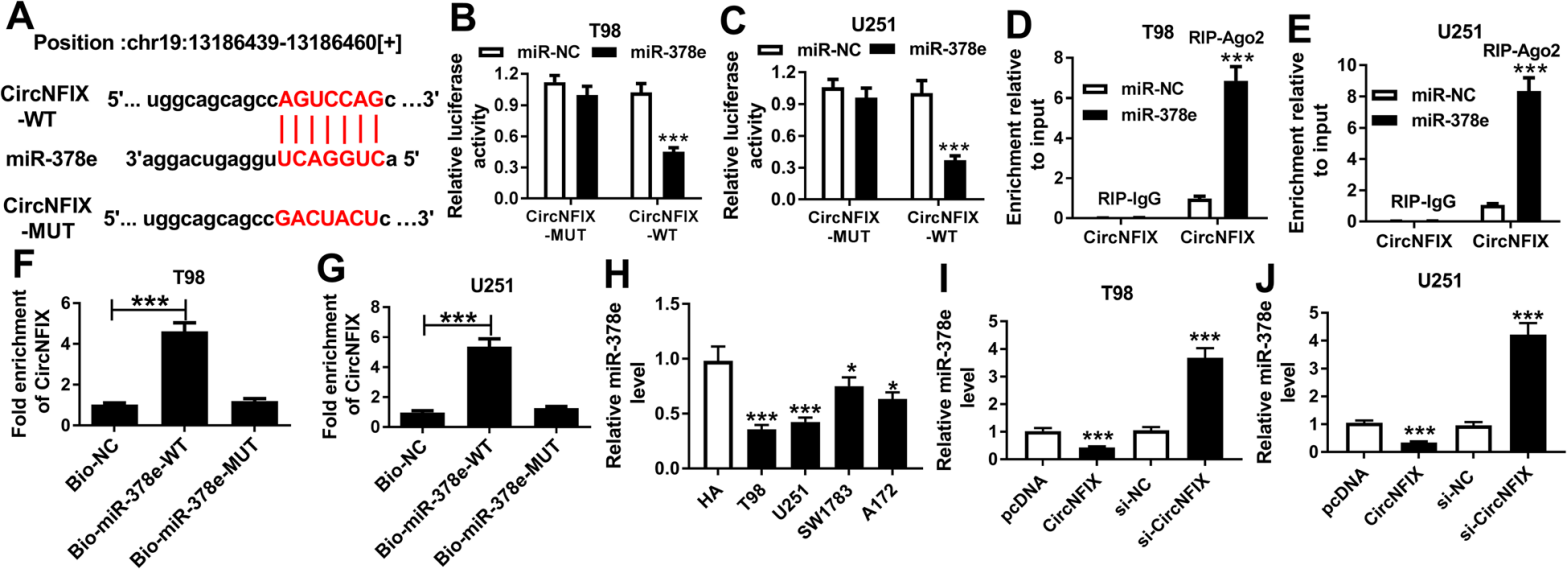
circNFIX is a sponge of miR-378e. (**a**) StarBase database predicted the binding sites of circNFIX and miR-378e. (**b-e**) Luciferase reporter assay and RIP assay were performed in T98 and U251 cells to confirm the association between circNFIX and miR-378e. (**f** and **g**) RNA pull-down assay was performed in T98 and U251 cells to confirm the association between circNFIX and miR-378e. (**h**) The expression level of miR-378e was measured in glioma cells and normal astrocyte cell line. (**i** and **j**) The abundances of miR-378e in T98 and U251 cells were detected after transfection of pcDNA, circNFIX, si-NC or si-circNFIX. *P < 0.05, ***P < 0.001 compared with indicated control group

This study detected the intracellular location of circNFIX and miR-378e and found that they were predominantly localized in the cytoplasm (Additional file 2: Figure S2A-S2D), indicating that circNFIX might act as a miRNA sponge. StarBase online predicted the targets of circNFIX and this database provided the complementary sequences between circNFIX and miR-378e at chr19: 13196439–13,186,460 (Fig. 3a). To confirm this association, circNFIX-WT and circNFIX-MUT were constructed and transfected into T98 and U251 cells. As shown in Fig. 3b and c, overexpression of miR-378e led to obvious loss of luciferase activity in circNFIX-WT group in the two cell lines, while it did not affect those in circNFIX-MUT group. Moreover, miR-378e overexpression led to higher enrichment level of circNFIX in Ago2 RIP group but not IgG RIP group (Fig. 3d and e). In addition, the data of RNA pull-down showed that there was abundant enrichment of circNFIX in Bio-miR-378e-WT group compared with that in Bio-NC group or Bio-miR-378e-MUT group (Fig. 3f and g). Furthermore, the level of miR-378e was detected in glioma cells and results showed that miR-378e abundance was aberrantly decreased in glioma cells in comparison to that in HA cells (Fig. 3h). Besides, qRT-PCR assay revealed that miR-378e abundance in T98 and U251 cells was evidently reduced by circNFIX overexpression and increased by circNFIX knockdown (Fig. 3i and j).

### circNFIX silence suppresses progression of glioma by regulating miR-378e

**Fig. 4:**
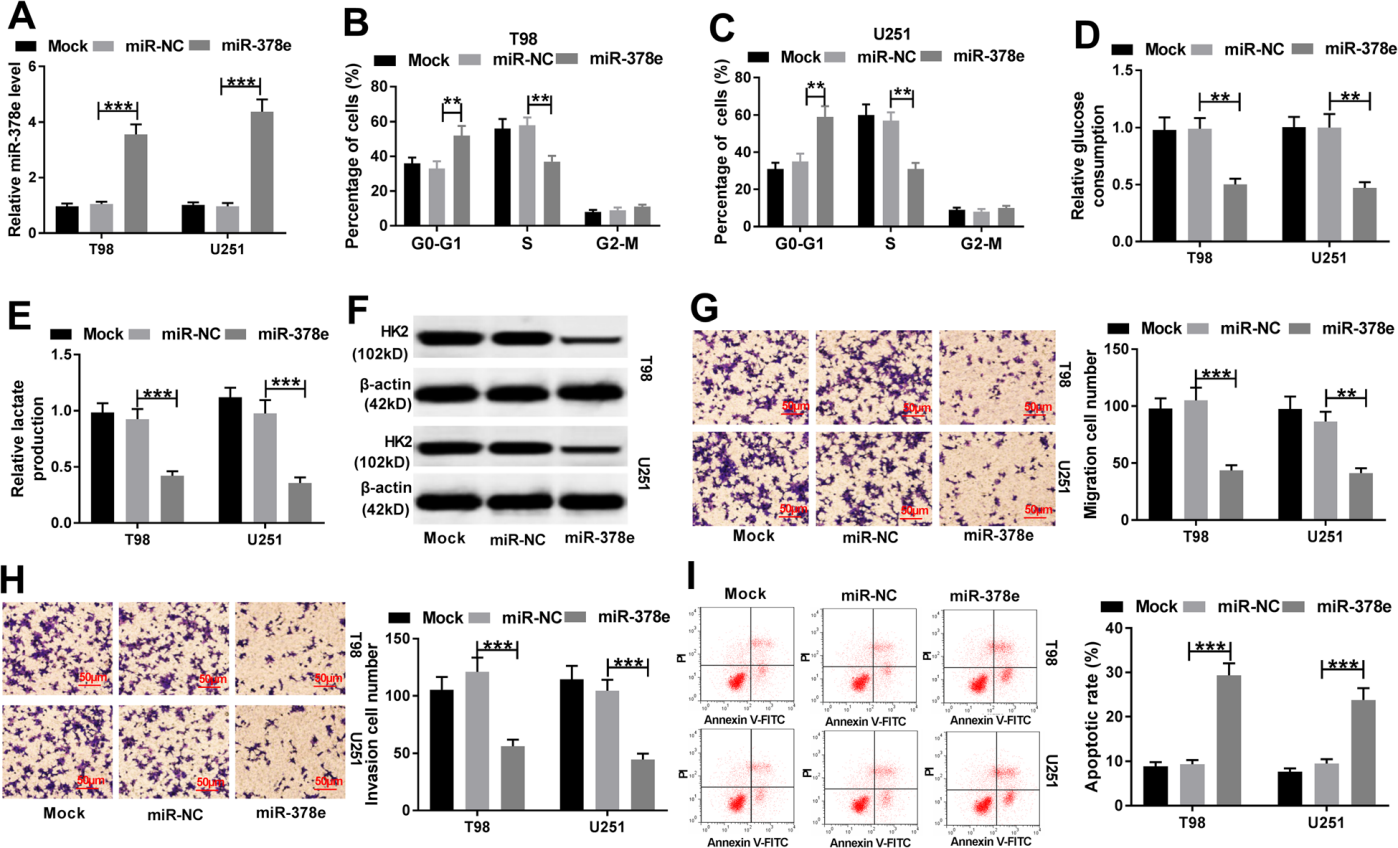
Overexpression of miR-378e induces cell cycle arrest and apoptosis and suppresses glycolysis, migration and invasion in glioma cells. (**a**) qRT-PCR assay was performed to detect the level of miR-378e in T98 and U251 cells after transfection of miR-378e or miR-NC. Cell cycle distribution (**b** and **c**), glucose consumption (**d**), lactate production (**e**), HK2 protein level (**f**), migration (**g**), invasion (**h**) and apoptosis (**i**) were examined in T98 and U251 cells transfected with miR-378e or miR-NC. Mock is non-transfected group. **P < 0.01, ***P < 0.001 compared with miR-NC group

**Fig. 5:**
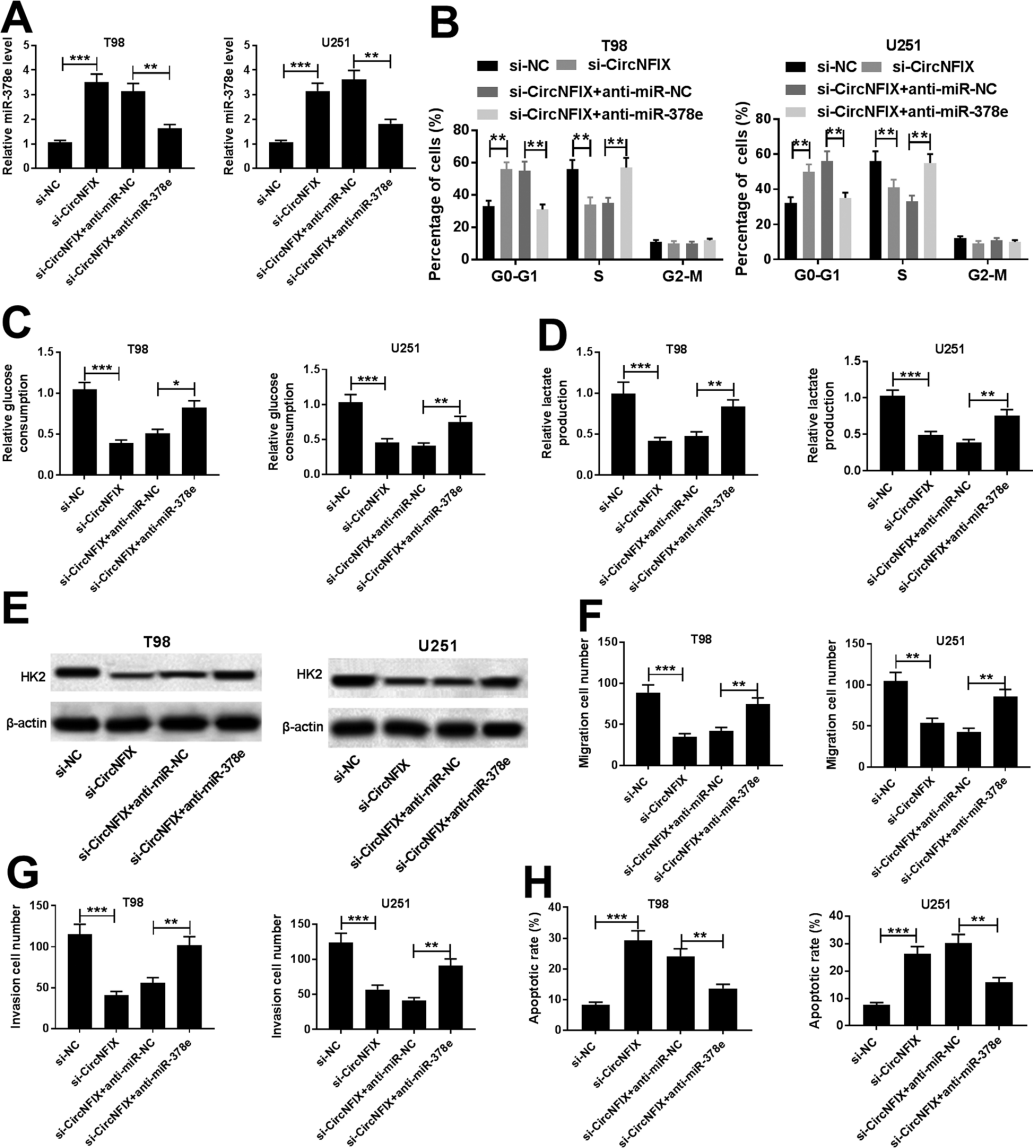
Deficiency of miR-378e attenuates the regulatory effect of circNFIX knockdown on cell cycle distribution, glycolysis, migration, invasion and apoptosis in glioma cells. miR-378e level (**a**), cell cycle distribution (**b**), glucose consumption (**c**), lactate production (**d**), HK2 protein level (**e**), migration (**f**), invasion (**g**) and apoptosis (**h**) were measured in T98 and U251 cells transfected with si-NC, si-circNFIX, si-circNFIX and anti-miR-NC or anti-miR-378e. *P < 0.05, **P < 0.01, ***P < 0.001 compared with indicated control group

Next, the role of miR-378e in glioma progression was investigated using T98 and U251 cells transfected with miR-378e or miR-NC. After the transfection, the level of miR-378e in T98 and U251 cells was effectively enhanced in miR-378e-transfected cells compared with that in miR-NC group (Fig. 4a). Furthermore, cell cycle of T98 and U251 cells was arrested at G0-G1 phase by miR-378e overexpression (Fig. 4b and c). Additionally, accumulation of miR-378e led to great reduction of glucose consumption, lactate production and HK2 protein expression in T98 and U251 cells (Fig. 4d-f). What’s more, addition of miR-378e remarkably blocked the abilities of migration and invasion in T98 and U251 cells (Fig. 4g and h). Moreover, apoptotic rate of T98 and U251 cells was significantly increased via miR-378e overexpression (Fig. 4i). To further explore whether miR-378e was involved in circNFIX-mediated glioma progression, T98 and U251 cells were transfected with si-NC, si-circNFIX, si-circNFIX and anti-miR-NC or anti-miR-378e. As shown in Fig. 5a, the level of miR-378e in the two cell lines was increased by circNFIX knockdown and it was obviously reduced by transfection of anti-miR-378e. Besides, cell cycle arrest, glycolysis inhibition, migration and invasion suppression and apoptosis production caused by silencing circNFIX were significantly attenuated by miR-378e exhaustion in T98 and U251 cells (Fig. 5b-h).

### circNFIX positively regulates RPN2 by sponging miR-378e in glioma cells

**Fig. 6:**
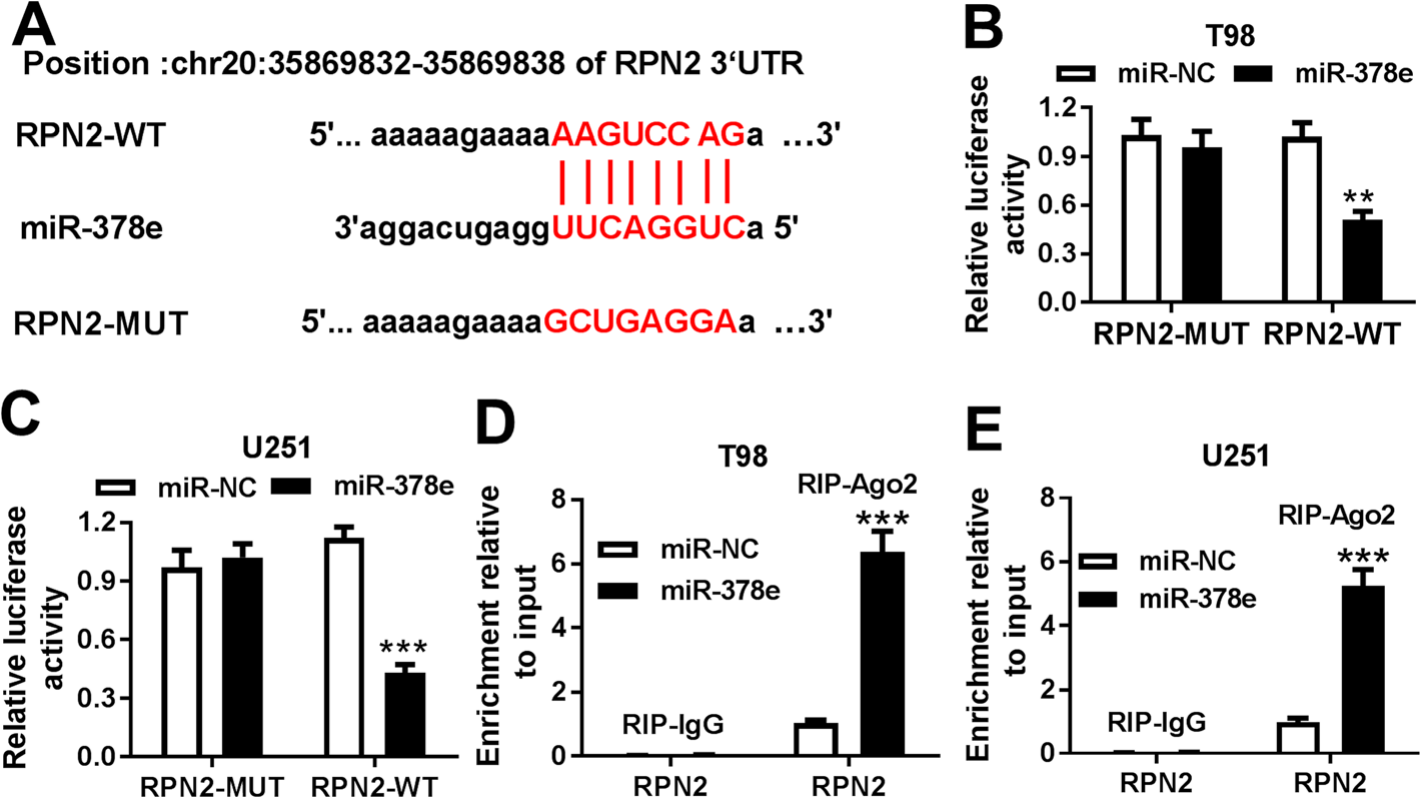
RPN2 is a target of miR-378e. (**a**) StarBase database predicted the complementary sequences between miR-378e and RPN2. (**b-e**) The target association between miR-378e and RPN2 in T98 and U251 cells was validated by luciferase reporter assay and RIP assay. **P < 0.01, ***P < 0.001 compared with miR-NC group

**Fig. 7:**
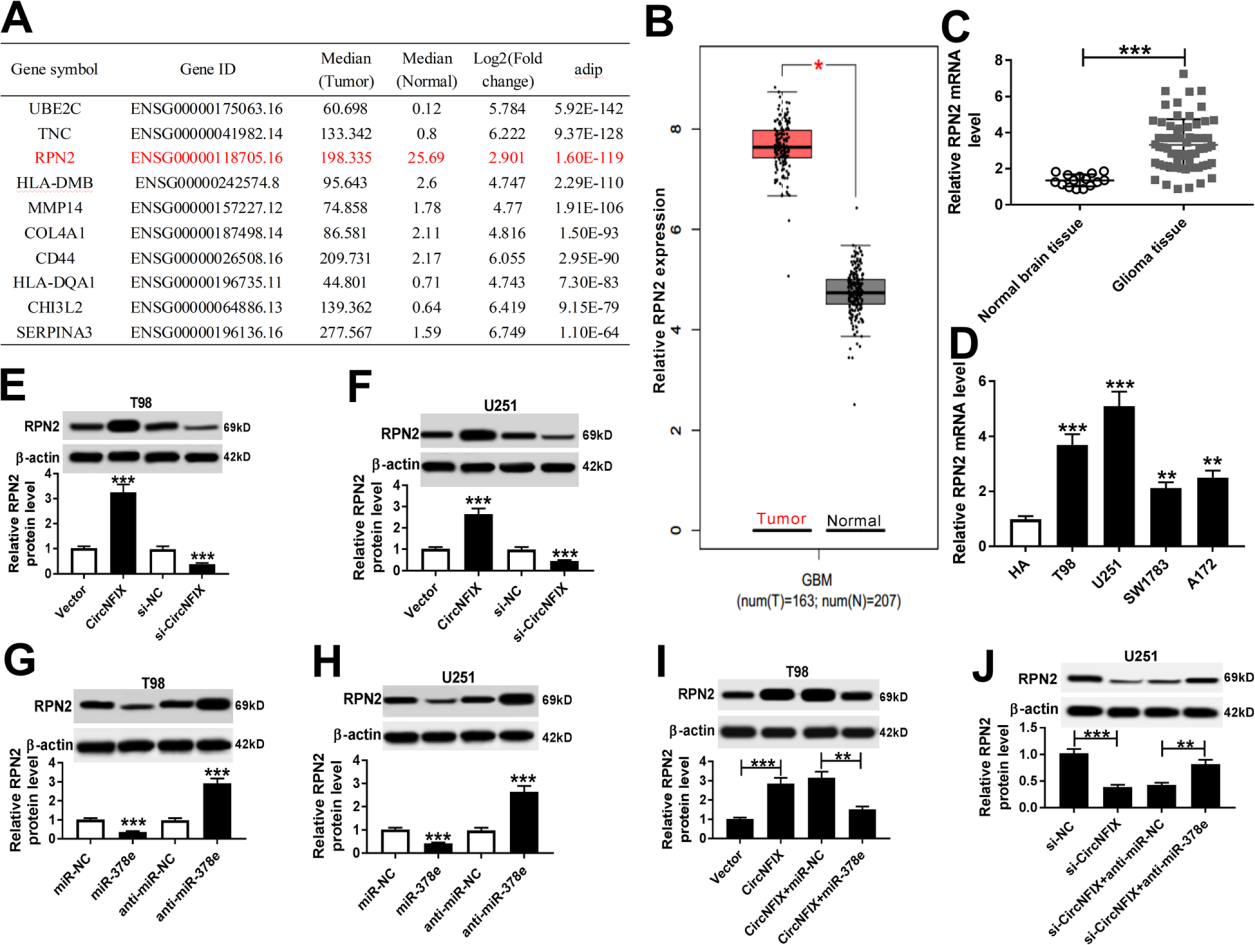
RPN2 is positively regulated by circNFIX and negatively regulated by miR-378e. (**a** and **b**) GERIA predicted high expression of RPN2 in glioma. (**c**) RPN2 mRNA level was detected in glioma tissues (*n* = 64) and normal tissues (*n* = 15). (**d**) The expression level of RPN2 mRNA was detected in glioma cells and normal astrocyte cell line. (**e** and **f**) RPN2 protein level was measured in T98 and U251 cells transfected with pcDNA, circNFIX, si-NC or si-circNFIX. (**g** and **h**) RPN2 protein level was detected in T98 and U251 cells transfected with miR-NC, miR-378e, anti-miR-NC or anti-miR-378e. (**i**) RPN2 protein level was determined in T98 and U251 cells transfected with pcDNA, circNFIX, circNFIX and miR-NC or miR-378e. (**j**) RPN2 protein level was determined in T98 and U251 cells transfected with si-NC, si-circNFIX, si-circNFIX and anti-miR-NC or anti-miR-378e. *P < 0.05, **P < 0.01, ***P < 0.001 compared with indicated control group

StarBase database predicted that RPN2 has the potential complementary sequences of miR-378e at chr20: 35869832–35,869,838 (Fig. 6a). Moreover, luciferase reporter assay was used to validate the target association with the results that miR-378e overexpression led to reduction of luciferase activity in RPN2-WT group, while its efficacy was lost when the seed sites were mutated (Fig. 6b and c). Meanwhile, miR-378e overexpression led to significantly increased RPN2 level in T98 and U251 cells after Ago2 RIP (Fig. 6d and e). In addition, GERIA online analyzed 10 abnormally expressed genes in glioma tissues, in which RPN2 was remarkably up-regulated in tumor tissues compared with that in normal group (Fig. 7a and b). Similarly, qRT-PCR assay in our study also displayed high expression of RPN2 in glioma tissues and cells compared with that in corresponding controls (Fig. 7c and d). Besides, the data of western blot described that RPN2 protein level in T98 and U251 cells was positively regulated by circNFIX (Fig. 7e and f) and negatively regulated by miR-378e (Fig. 7g and h). Meanwhile, circNFIX-mediated RPN2 protein expression was counteracted by introduction of miR-378e (Fig. 7i and j).

### Silence of circNFIX decreases glioma xenograft tumor growth by regulating miR-378e/RPN2 axis

**Fig. 8:**
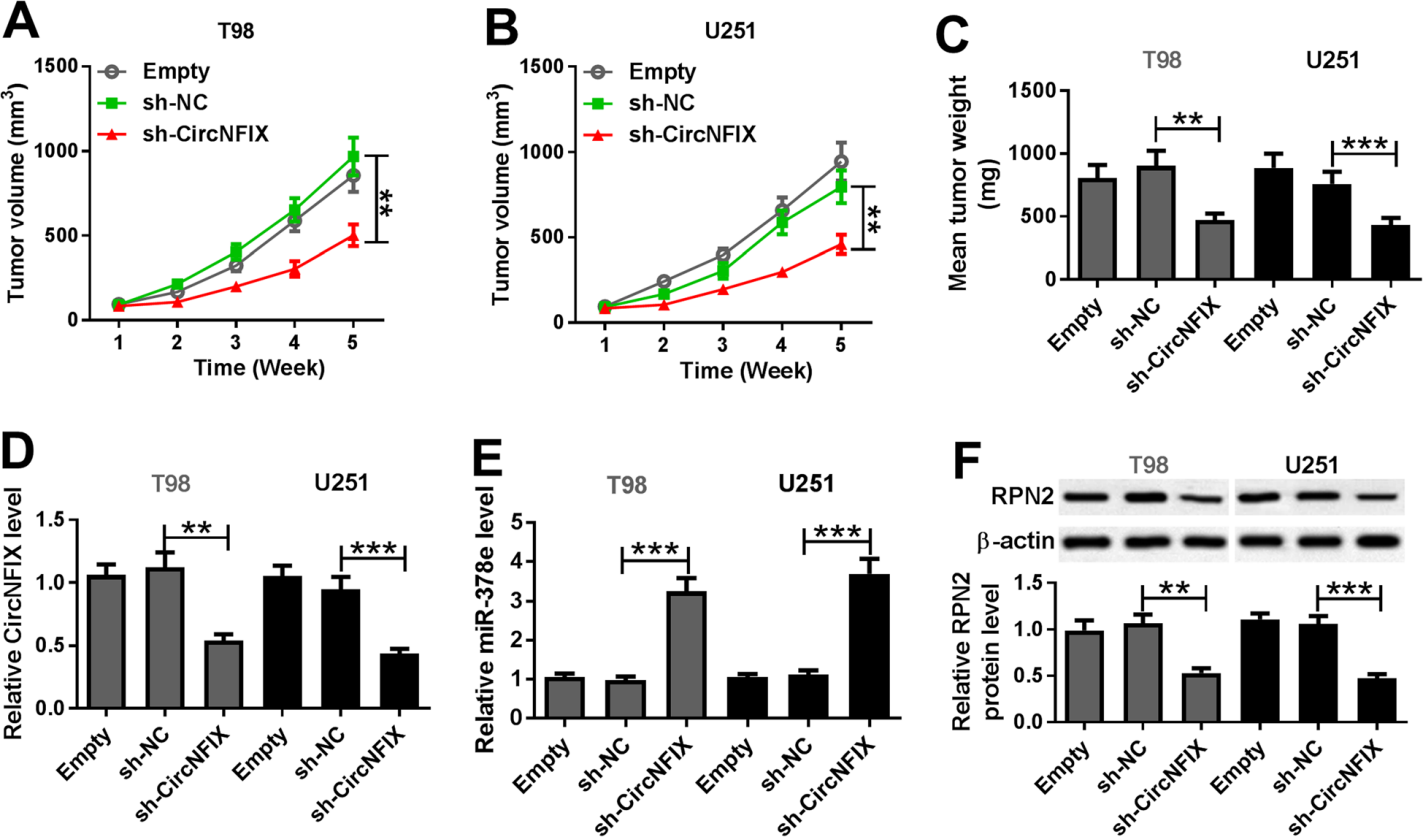
Silence of circNFIX decreases xenograft tumor growth by regulating miR-378e/RPN2 axis. T98 and U251 cells were stably transfected with sh-NC or sh-circNFIX and then infected into nude mice. (**a-c**) Tumor volume and weight were measured. (**d-f**) The expression levels of circNFIX, miR-378e and RPN2 protein were measured in tumor tissues. **P < 0.01, ***P < 0.001 compared with sh-NC group

To further explore the biological role of circNFIX in glioma in vivo, T98 and U251 cells stably transfected with sh-circNFIX or sh-NC were injected into nude mice to establish xenograft model. As shown in Fig. 8a and b, the volume of tumor induced by T98 and U251 xenograft was obviously decreased in sh-circNFIX group compared with that in sh-NC group. Meanwhile, the tumor weight was markedly reduced in sh-circNFIX group when compared with sh-NC group (Fig. 8c). Moreover, the tumor tissues were collected and the levels of circNFIX, miR-378e and RPN2 were detected. As displayed in Fig. 8d-f, the levels of circNFIX and RPN2 protein were notably decreased but miR-378e level was enhanced in sh-circNFIX group compared with those in sh-NC group.

## Discussion

Previous study displayed that circRNAs have essential roles in the development and treatment of glioma [5]. However, much is still unknown about the function of many circRNAs. The analysis of qRT-PCR in this study presented that circNFIX was up-regulated in glioma, suggesting that this circRNA might play the promoting role in glioma progression. The focus of this project was to analyze the anti-cancer role of circNFIX in glioma and explore the novel ceRNA network of circNFIX/miR-378e/RPN2.

Our study showed that silencing circNFIX inhibited migration and invasion but promoted apoptosis in glioma, which is also consistent with former work [12]. Normal cell cycle allows cell to grow but arrest of cell cycle could lead to cell death [20]. In this study, we found that cell apoptosis induced by circNFIX knockdown might be associated with cell cycle arrest at G0-G1 phase. Moreover, glycolysis is an important way to maintain cell survival in cancers including glioma and lactate is a key product as a biomarker of malignancy [21–23]. And HK2 is a key enzyme during glucose metabolism in glycolysis [24]. By detecting glucose consumption, lactate production and HK2 protein level, we were the first to provide that circNFIX knockdown decreased glycolysis in glioma. These findings uncovered the tumor suppressive role of circNFIX inhibition in glioma.

Moreover, we studied the function of miRNA in glioma progression affected via circNFIX. We first used luciferase reporter, RIP and RNA pull-down assays to validate the target association between circNFIX and miR-378e. Usually, circRNAs could act as sponges of miRNAs to regulate their activity. However, this study showed that miR-378e expression was decreased by circNT5E. We hypothesized that might be induced by the digestion of proteins in the circRNA-protein complex. miR-378 has been reported as a tumor suppressor in colon cancer and pituitary adenoma [25, 26], while as oncogenic miRNA in osteosarcoma, cervical cancer and cholangiocarcinoma [27–29]. We hypothesized the different function might be induced by various tumor microenvironment. Moreover, decreased expression of miR-378 indicated poor prognosis and its overexpression suppressed migration, invasion and epithelial-mesenchymal transition in glioma [15, 16]. In this research, we also found the suppressive role of miR-378e in migration and invasion of glioma cells. Meanwhile, this work showed that miR-378e overexpression inhibited glycolysis and promoted cell apoptosis, indicating the therapeutic effect of miR-378e in glioma. Additionally, rescue experiments by transfection of miR-378e inhibitor in the presence of si-circNFIX uncovered that circNFIX regulated glioma progression by sponging miR-378e.

Next, targets of miR-378e were explored and here we first confirmed RPN2 as a functional target of miR-378e in glioma. Accruing studies suggested RPN2 as an oncogene in multiple cancers, including breast cancer, colon carcinoma, nasopharyngeal carcinoma and esophageal cancer [30–33]. Hence, we hypothesized that RPN2 might be also as a carcinogenic gene in glioma. By detecting its level in glioma tissues and cells, we found that RPN2 was highly expressed in glioma, which is also in agreement with the data of GERIA, indicating that high expression of RPN2 might contribute to glioma development, although the exact role of RPN2 in glioma was not investigated in the current research. Moreover, RPN2 was predicted with the same miR-378e binding sites with circNFIX, suggesting the potential ceRNA of circNFIX. This study using western blot assay validated that circNFIX could promote RPN2 expression by competitively sponging miR-378e, uncovering the ceRNA network of circNFIX/miR-378e/RPN2 in glioma. Xenograft model is responsible for the preclinical trials in glioma pathogenesis [34]. Furthermore, we used T98 and U251 xenograft model to confirm the anti-glioma role of circNFIX inhibition in vivo. The xenograft model includes orthotopic tumor and subcutaneous tumor. In this study, we used subcutaneous model to investigate the role of circNFIX in glioma in vivo. During 5 weeks of observation, no mouse died. We hypothesized that it might be resulted from the limitation of cancer cell diffusion and metastasis in subcutaneous locations. Moreover, the more cell number and longer growth time might also affect the survival of mice. Besides, the exact role of RPN2 in glioma progression was absent in the present study, which should be explored in future. Moreover, previous studies suggested that PRN2 could activate extracellular regulated protein kinase (ERK) signaling and this pathway was associated with glioma progression [35, 36]. In the current work, we also found that PRN2 promoted the activation of ERK pathway in glioma cells (Additional file 3: Figure S3A and S3B). However, the direct evidence between ERK signaling and circNFIX-mediated glioma progression was absent, which is expected to be explored in further work.

## Conclusion

In conclusion, our study on the oncogenic role of circNFIX in glioma showed that knockdown of circNFIX suppressed progression of glioma in vitro and in vivo, possibly by regulating miR-378e/RPN2 axis as a ceRNA. This study elucidated a new mechanism for development of glioma and indicated a novel target for treatment of glioma.

## Supplementary information

**Additional file 1.** Supplementary materials and methods. Subcellular fraction assay. The separation of cytoplasmic and nuclear fractions was performed using Nuclear and Cytoplasmic Extraction Reagents (Thermo Fisher Scientific, Wilmington, DE, USA) following the manufacturer’s protocols. Total RNA extracted from each fraction was used for detection of circNFIX and miR-378e by qRT-PCR assay. The relative expression levels of circNFIX and miR-378e in cytoplasmic and nuclear fractions were analyzed using GAPDH or U6 as cytoplasmic or nuclear control, respectively.

**Additional file 2: Figure S1.** CircNFIX was more resistant to RNase R than NFIX in glioma cells. (A) NFIX was a host gene of circNFIX (hsa_circ_0049658) and sanger sequencing validated the sequence on the junction sites of circNFIX. (B and C) The expression levels of circNFIX and NFIX were measured in U251 and T98 cells after treatment of RNase R. ***P<0.001. **Figure S2.** CircNFIX and miR-378e were predominantly localized in the cytoplasm. (A-D) The expression levels of circNFIX and miR-378e were measured in cytoplasmic and nuclear fractions, with GAPDH and U6 as the internal controls, respectively. **Figure S3.** RPN2 promoted the activation of ERK pathway in glioma cells. (A and B) The protein levels of p-ERK, ERK, p=MEK and MEK were measured in U251 cells transfected with pcDNA or RPN2 and T98 cells transfected with si-NC or si-RPN2. ***P<0.001.

## Data Availability

Data sharing not applicable to this article as no datasets were generated or analyzed during the current study.

## Abbreviations

ERK: Extracellular regulated protein kinase
NFIX: Nuclear factor I X
PI3K: Phosphatidylinositol 3 kinase
RPN2: CircNFIX/Ribophorin-II
SIRT1: Silent information regulator 1

## References

[1] Ghotme KA, Barreto GE, Echeverria V, Gonzalez J, Bustos RH, Sanchez M (2017). Gliomas: new perspectives in diagnosis, treatment and prognosis. Curr Top Med Chem.

[2] Wang D, Yang S, Wang H, Wang J, Zhang Q, Zhou S (2018). The progress of circular RNAs in various tumors. Am J Transl Res.

[3] Qu S, Yang X, Li X, Wang J, Gao Y, Shang R (2015). Circular RNA: a new star of noncoding RNAs. Cancer Lett.

[4] Gokul S, Rajanikant GK (2018). Circular RNAs in brain physiology and disease. Adv Exp Med Biol.

[5] Hao Z, Hu S, Liu Z, Song W, Zhao Y, Li M (2019). Circular RNAs: functions and prospects in Glioma. J Mol Neurosci.

[6] Lei B, Huang Y, Zhou Z, Zhao Y, Thapa AJ, Li W (2019). Circular RNA hsa_circ_0076248 promotes oncogenesis of glioma by sponging miR-181a to modulate SIRT1 expression. J Cell Biochem.

[7] Shi F, Shi Z, Zhao Y, Tian J (2019). CircRNA hsa-circ-0014359 promotes glioma progression by regulating miR-153/PI3K signaling. Biochem Biophys Res Commun.

[8] Wang J, Li J, Wang H, Lv L, Sun J (2019). Overexpression of circ_0005198 sponges miR-1294 to regulate cell proliferation, apoptosis, migration, and invasion in glioma. J Cell Biochem.

[9] Qu Y, Zhu J, Liu J, Qi L (2019). Circular RNA circ_0079593 indicates a poor prognosis and facilitates cell growth and invasion by sponging miR-182 and miR-433 in glioma. J Cell Biochem.

[10] Piper M, Gronostajski R, Messina G (2019). Nuclear factor one X in development and disease. Trends Cell Biol.

[11] Campbell CE, Piper M, Plachez C, Yeh YT, Baizer JS, Osinski JM (2008). The transcription factor Nfix is essential for normal brain development. BMC Dev Biol.

[12] Xu H, Zhang Y, Qi L, Ding L, Jiang H, Yu H (2018). NFIX circular RNA promotes Glioma progression by regulating miR-34a-5p via notch signaling pathway. Front Mol Neurosci.

[13] Petrescu GED, Sabo AA, Torsin LI, Calin GA, Dragomir MP (2019). MicroRNA based theranostics for brain cancer: basic principles. J Exp Clin Cancer Res.

[14] Wang S, Yin Y, Liu S (2019). Roles of microRNAs during glioma tumorigenesis and progression. Histol Histopathol.

[15] Li B, Wang Y, Li S, He H, Sun F, Wang C (2015). Decreased expression of miR-378 correlates with tumor invasiveness and poor prognosis of patients with glioma. Int J Clin Exp Pathol.

[16] Shi HZ, Wang D, Sun XN, Sheng L (2018). MicroRNA-378 acts as a prognosis marker and inhibits cell migration, invasion and epithelial-mesenchymal transition in human glioma by targeting IRG1. Eur Rev Med Pharmacol Sci.

[17] Zhong Y, Du Y, Yang X, Mo Y, Fan C, Xiong F (2018). Circular RNAs function as ceRNAs to regulate and control human cancer progression. Mol Cancer.

[18] Komorowski M, Marshall DC, Salciccioli JD, Crutain Y (2016). Exploratory data analysis.

[19] Livak KJ, Schmittgen TD (2001). Analysis of relative gene expression data using real-time quantitative PCR and the 2(−Delta Delta C(T)) method. Methods.

[20] Schwartz GK, Shah MA (2005). Targeting the cell cycle: a new approach to cancer therapy. J Clin Oncol.

[21] Vander Heiden MG, Cantley LC, Thompson CB (2009). Understanding the Warburg effect: the metabolic requirements of cell proliferation. Science.

[22] Liu X, Zhu Q, Guo Y, Xiao Z, Hu L, Xu Q (2019). LncRNA LINC00689 promotes the growth, metastasis and glycolysis of glioma cells by targeting miR-338-3p/PKM2 axis. Biomed Pharmacother.

[23] Ippolito L, Morandi A, Giannoni E, Chiarugi P (2019). Lactate: a metabolic driver in the tumour landscape. Trends Biochem Sci.

[24] Tan VP, Miyamoto S (2015). HK2/hexokinase-II integrates glycolysis and autophagy to confer cellular protection. Autophagy.

[25] Zeng M, Zhu L, Li L, Kang C (2017). miR-378 suppresses the proliferation, migration and invasion of colon cancer cells by inhibiting SDAD1. Cell Mol Biol Lett.

[26] Qiu P, Xu TJ, Lu XD, Yang W, Zhang YB, Xu GM (2018). MicroRNA-378 regulates cell proliferation and migration by repressing RNF31 in pituitary adenoma. Oncol Lett.

[27] Peng N, Miao Z, Wang L, Liu B, Wang G, Guo X (2018). MiR-378 promotes the cell proliferation of osteosarcoma through down-regulating the expression of Kruppel-like factor 9. Biochem Cell Biol.

[28] Tan D, Zhou C, Han S, Hou X, Kang S, Zhang Y (2018). MicroRNA-378 enhances migration and invasion in cervical cancer by directly targeting autophagy-related protein 12. Mol Med Rep.

[29] Zhou Z, Ma J (2019). miR-378 serves as a prognostic biomarker in cholangiocarcinoma and promotes tumor proliferation, migration, and invasion. Cancer Biomark.

[30] Ono M, Tsuda H, Kobayashi T, Takeshita F, Takahashi RU, Tamura K (2015). The expression and clinical significance of ribophorin II (RPN2) in human breast cancer. Pathol Int.

[31] Bi C, Jiang B (2018). Downregulation of RPN2 induces apoptosis and inhibits migration and invasion in colon carcinoma. Oncol Rep.

[32] Hong F, Li Y, Ni H, Li J (2018). Downregulation of ribophorin II suppresses tumor growth, migration, and invasion of nasopharyngeal carcinoma. Onco Targets Ther.

[33] Li Y, Huang C, Bai Q, Yu J (2019). Ribophorin II promotes cell proliferation, migration, and invasion in esophageal cancer cells in vitro and in vivo. Biosci Rep.

[34] Chen L, Zhang Y, Yang J, Hagan JP, Li M (1836). Vertebrate animal models of glioma: understanding the mechanisms and developing new therapies. Biochim Biophys Acta.

[35] Zhang H, Jiang H, Zhang H, Liu J, Hu X, Chen L (2019). Ribophorin II potentiates P-glycoprotein- and ABCG2-mediated multidrug resistance via activating ERK pathway in gastric cancer. Int J Biol Macromol.

[36] Guo G, Yao W, Zhang Q, Bo Y (2013). Oleanolic acid suppresses migration and invasion of malignant glioma cells by inactivating MAPK/ERK signaling pathway. PLoS One.

