# Supplementary material for: CircNFIX promotes progression of glioma through regulating miR-378e/RPN2 axis
Source: J Exp Clin Cancer Res. 2019 Dec 30;38:506. doi: 10.1186/s13046-019-1483-6 (PMC6936104; doi:10.1186/s13046-019-1483-6)
Supplement: Supplementary file 1 — Additional file 1. Supplementary materials and methods. Subcellular fraction assay. The separation of cytoplasmic and nuclear fractions was performed using Nuclear and Cytoplasmic Extraction Reagents (Thermo Fisher Scientific, Wilmington, DE, USA) following the manufacturer’s protocols. Total RNA extracted from each fraction was used for detection of circNFIX and miR-378e by qRT-PCR assay. The relative expression levels of circNFIX and miR-378e in cytoplasmic and nuclear fractions were analyzed using GAPDH or U6 as cytoplasmic or nuclear control, respectively. [file 13046_2019_1483_MOESM1_ESM.docx]

**Supplementary materials and methods**

**Subcellular fraction assay**

The separation of cytoplasmic and nuclear fractions was performed using Nuclear and Cytoplasmic Extraction Reagents (Thermo Fisher Scientific, Wilmington, DE, USA) following the manufacturer’s protocols. Total RNA extracted from each fraction was used for detection of circNFIX and miR-378e by qRT-PCR assay. The relative expression levels of circNFIX and miR-378e in cytoplasmic and nuclear fractions were analyzed using GAPDH or U6 as cytoplasmic or nuclear control, respectively.
