# Supplementary figures and images for: CircNFIX promotes progression of glioma through regulating miR-378e/RPN2 axis
Source: J Exp Clin Cancer Res. 2019 Dec 30;38:506. doi: 10.1186/s13046-019-1483-6 (PMC6936104; doi:10.1186/s13046-019-1483-6)

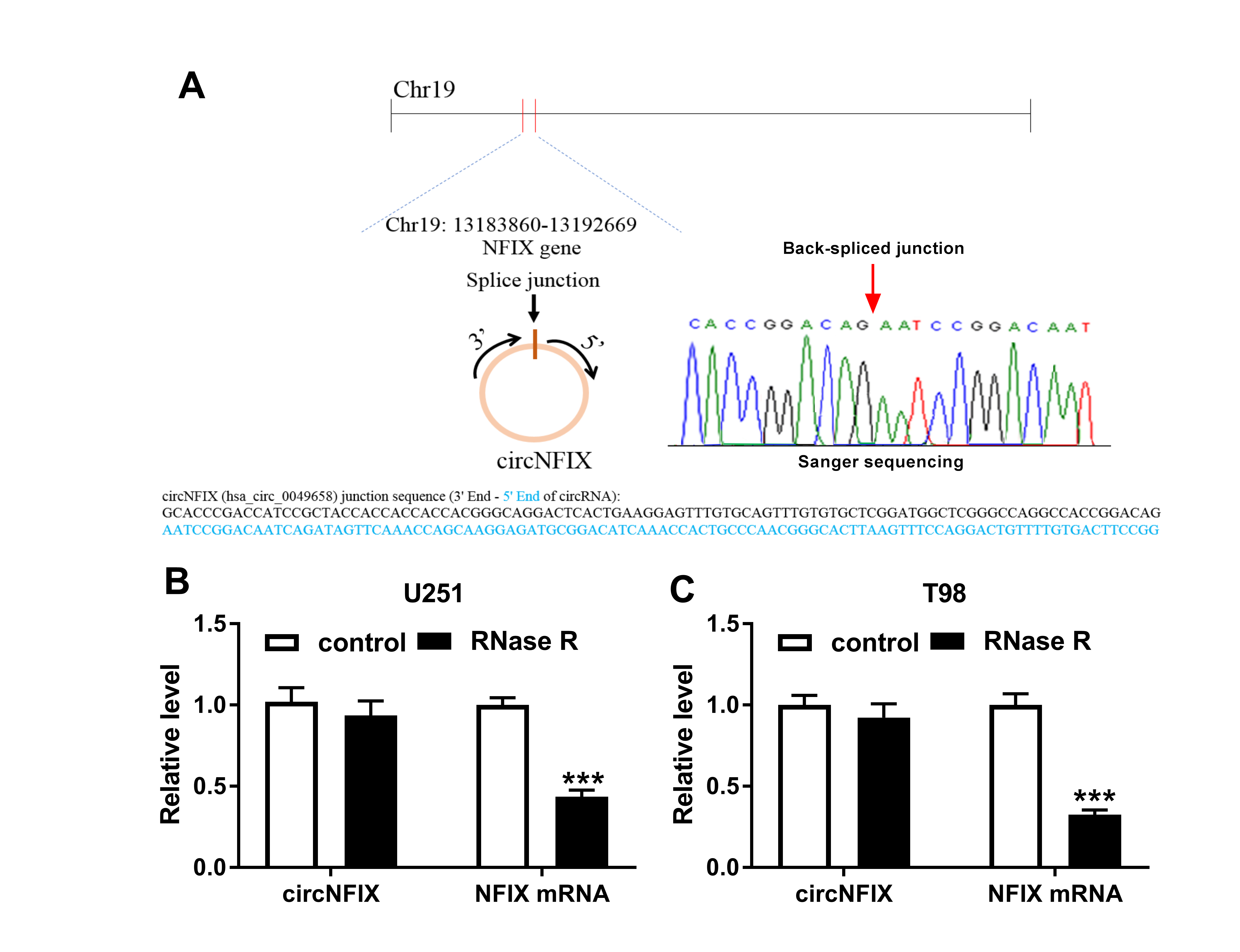

Supplement: Supplementary file 2 — Additional file 2: Figure S1. CircNFIX was more resistant to RNase R than NFIX in glioma cells. (A) NFIX was a host gene of circNFIX (hsa_circ_0049658) and sanger sequencing validated the sequence on the junction sites of circNFIX. (B and C) The expression levels of circNFIX and NFIX were measured in U251 and T98 cells after treatment of RNase R. ***P<0.001. Figure S2. CircNFIX and miR-378e were predominantly localized in the cytoplasm. (A-D) The expression levels of circNFIX and miR-378e were measured in cytoplasmic and nuclear fractions, with GAPDH and U6 as the internal controls, respectively. Figure S3. RPN2 promoted the activation of ERK pathway in glioma cells. (A and B) The protein levels of p-ERK, ERK, p=MEK and MEK were measured in U251 cells transfected with pcDNA or RPN2 and T98 cells transfected with si-NC or si-RPN2. ***P<0.001. [file 13046_2019_1483_MOESM2_ESM.zip › revised Sup Fig 1.tif]

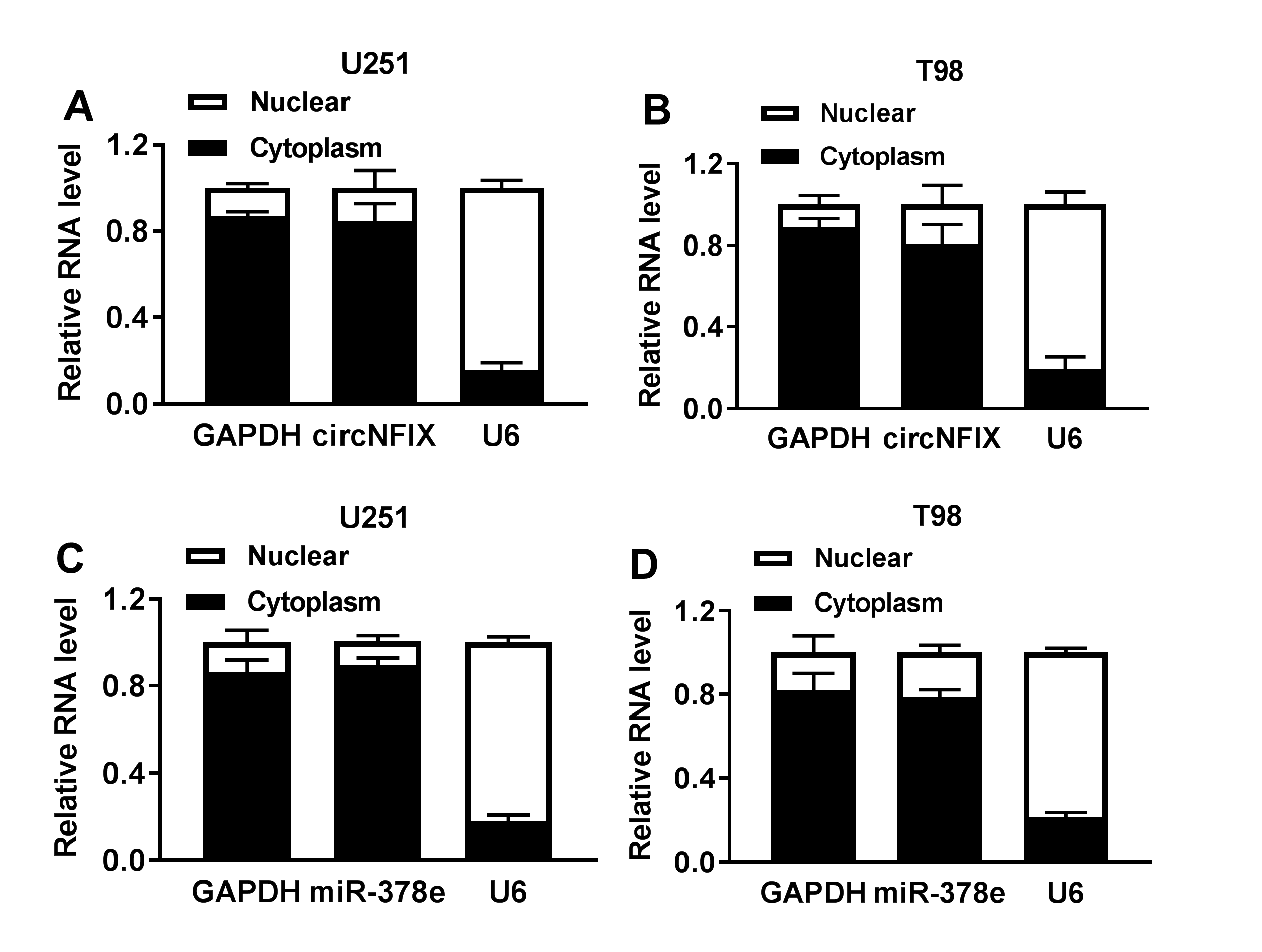

Supplement: Supplementary file 2 — Additional file 2: Figure S1. CircNFIX was more resistant to RNase R than NFIX in glioma cells. (A) NFIX was a host gene of circNFIX (hsa_circ_0049658) and sanger sequencing validated the sequence on the junction sites of circNFIX. (B and C) The expression levels of circNFIX and NFIX were measured in U251 and T98 cells after treatment of RNase R. ***P<0.001. Figure S2. CircNFIX and miR-378e were predominantly localized in the cytoplasm. (A-D) The expression levels of circNFIX and miR-378e were measured in cytoplasmic and nuclear fractions, with GAPDH and U6 as the internal controls, respectively. Figure S3. RPN2 promoted the activation of ERK pathway in glioma cells. (A and B) The protein levels of p-ERK, ERK, p=MEK and MEK were measured in U251 cells transfected with pcDNA or RPN2 and T98 cells transfected with si-NC or si-RPN2. ***P<0.001. [file 13046_2019_1483_MOESM2_ESM.zip › supplementary figure 2.tif]

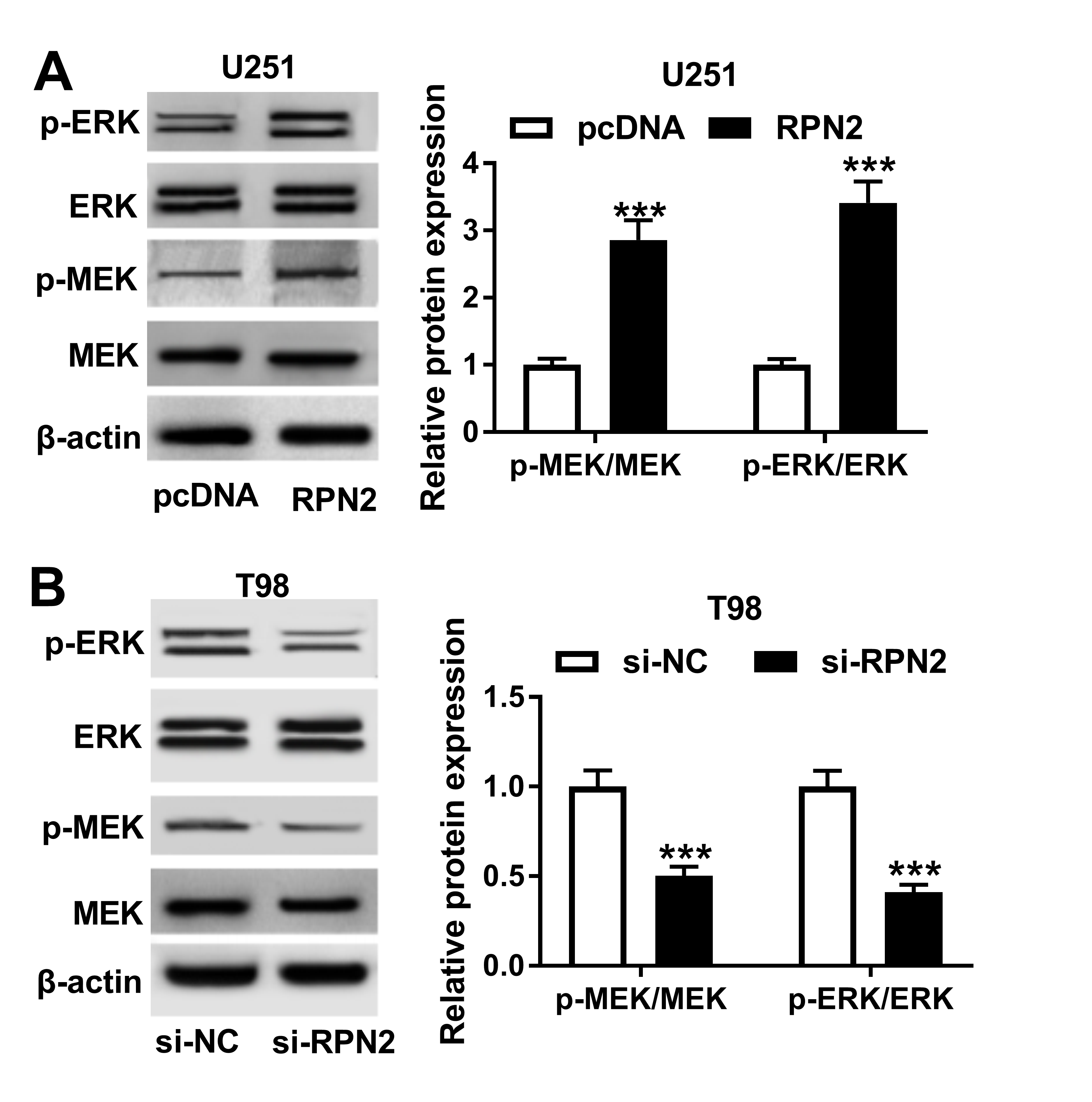

Supplement: Supplementary file 2 — Additional file 2: Figure S1. CircNFIX was more resistant to RNase R than NFIX in glioma cells. (A) NFIX was a host gene of circNFIX (hsa_circ_0049658) and sanger sequencing validated the sequence on the junction sites of circNFIX. (B and C) The expression levels of circNFIX and NFIX were measured in U251 and T98 cells after treatment of RNase R. ***P<0.001. Figure S2. CircNFIX and miR-378e were predominantly localized in the cytoplasm. (A-D) The expression levels of circNFIX and miR-378e were measured in cytoplasmic and nuclear fractions, with GAPDH and U6 as the internal controls, respectively. Figure S3. RPN2 promoted the activation of ERK pathway in glioma cells. (A and B) The protein levels of p-ERK, ERK, p=MEK and MEK were measured in U251 cells transfected with pcDNA or RPN2 and T98 cells transfected with si-NC or si-RPN2. ***P<0.001. [file 13046_2019_1483_MOESM2_ESM.zip › supplementary figure 3.tif]
